# Editorial: Current molecular, immunological, pathological and clinical aspects of pathogenic infections

**DOI:** 10.3389/fcimb.2025.1665283

**Published:** 2025-08-08

**Authors:** José Luis Muñoz-Carrillo, Oscar Gutiérrez-Coronado, Rommel Chacon-Salinas

**Affiliations:** ^1^ Laboratorio de Inmunología, Centro Universitario de los Lagos, Universidad de Guadalajara, Lagos de Moreno, Jalisco, Mexico; ^2^ Escuela de Odontología, Global University, Aguascalientes, Aguascalientes, Mexico; ^3^ Departamento de Inmunología, Escuela Nacional de Ciencias Biológicas, Instituto Politécnico Nacional, Ciudad de Mexico, Mexico

**Keywords:** bovine respiratory disease, COVID-19, hepatitis B virus, mycoplasma pneumoniae, periodontitis, puerperal infection, sepsis, type 2 diabetes mellitus

Infectious diseases represent a significant risk to human health and have caused approximately more than half of all deaths worldwide (Muñoz-Carrillo et al., 2024). However, the human body possesses mechanisms capable of recognizing and identifying infections caused by pathogens and defending the organism through the early reactions of innate immunity and the late reactions of adaptive immunity (Wang et al., 2024). The innate immune response recognizes pathogen-associated molecular patterns (PAMPs), these PAMPs are recognized by pattern recognition receptors (PRRs), expressed mainly in innate immunity cells; while the adaptive immune response is antigen-specific. In both responses, several immune system cells are activated, playing a key role in establishing the cytokine environment, thereby directing their differentiation, either suppressing or promoting the immune response, which is crucial against pathogen infections (Muñoz-Carrillo et al., 2018). The immune system consists of a series of effector mechanisms capable of destroying pathogenic organisms such as bacteria, fungi, viruses and parasites. These mechanisms consist of (1) preventing the entry of pathogens into the body through physical and chemical barriers; (2) preventing the spread of infections through the complement system and other humoral factors; (3) eliminating pathogens through phagocytosis and cytotoxicity; and (4) activating the adaptive immune system through the synthesis of various cytokines and the presentation of antigens to T and B cells (Muñoz Carrillo et al., 2017). However, sometimes the immune system cannot resolve the various diseases caused by pathogens. Therefore, some type of therapy, whether pharmacological, immunological, etc., is necessary to resolve the disease.

Because diseases caused by pathogens are currently of great interest and importance in the biomedical field, due to the impact they have on the health of the human population, the main aim of this Research Topic was to show recent advances in molecular, immunological, pathological and clinical aspects of infections and diseases caused by these pathogens. In this context, eleven articles were published in this Research Topic: nine of them were original research articles, one narrative review and one mini review. The most relevant findings from each are described below.

Puerperal infection (PI), responsible for 11% of maternal deaths worldwide, and it is a preventable cause of morbidity and mortality. In the study by Wen et al., the main risk factors and pathogenic bacteria associated with IP were analyzed in 525 women, through a validated predictive model with high precision with an area under the ROC curve of 0.904 (95% CI: 0.871-0.936) in the training set and 0.890 (95% CI: 0.837-0.942) in the test set. Six significant risk factors (*p<0.05) were identified: parity, number of vaginal examinations, amount of postpartum bleeding, antibiotics administered one week before admission, induced labor, and indwelling catheter. On the other hand, drug sensitivity map showed that *Escherichia coli* was the main pathogen (89%), with high sensitivity to antibiotics such as Meropenem and Imipenem (100%), Piperacillin tazobactam (97.7%), Ceftazidime (95.5%), and Amoxicillin/Clavulanate (AMC, 93.3%). These findings provide useful guidance for clinical prevention and treatment, reducing the risk of PI by controlling the number of vaginal examinations, postpartum bleeding, and reducing the time of urethral catheter indwelling, with the recommendation to use antibiotics sensitive to *Escherichia coli*.

Bacteremia is the presence of viable bacteria in the blood, a potentially life-threatening condition that can progress to sepsis, characterized primarily by a systemic inflammatory response of the host to bacterial infection. Bacteremia is responsible for approximately 8 million deaths per year worldwide, and the most common pathogenic bacteria involved are *Escherichia coli*, *Klebsiella pneumoniae*, and *Staphylococcus aureus* (Font et al., 2020; Xu et al., 2024). In the study by Zhang et al., the distribution of bacteremia pathogens in elderly patients (≥60 years old) and the impact of gender on this were evaluated, as well as the predictive value of routine blood parameters for diagnosis bacteremia. This study revealed that the main pathogens of bacteremia were *Escherichia coli*, *Klebsiella pneumoniae* and *Streptococcus*, with *Escherichia coli* being significantly (*p=0.021*) more frequent in elderly female patients. Furthermore, analysis of the area under the ROC curve showed that the most effective parameters for predicting bacteremia were the leukocyte count [0.851(95% CI: 0.790 - 0.912)] and the neutrophil-lymphocyte ratio [0.919 (95% CI 0.875 - 0.963)]. These findings support the use of routine blood parameters as useful tools for the early diagnosis of bacteremia in elderly patients.

Periprosthetic joint infection (PJI) is a severe complication characterized by high morbidity, mortality (Abedi et al., 2025), and resistance to antimicrobial treatment (Wouthuyzen-Bakker et al., 2019; Ryan et al., 2024), mainly due to the formation of bacterial biofilms on prosthetic materials, generating chronic infections (Edmiston et al., 2015). These biofilms make it difficult to eradicate the infection, requiring prolonged antibiotic treatment and surgery, including implant removal (Visperas et al., 2022). However, these interventions involve high costs and limited clinical results (Xu et al., 2023). It is noteworthy that factors such as obesity and diabetes increase the incidence of PJI (Kapadia et al., 2016), with obesity being a significant risk factor for treatment failure (Houdek et al., 2015; Watts et al., 2015). In the study by Shi et al., the impact of obesity on the diagnosis of PJI was evaluated through the quantification of levels of biomarkers [C-reactive protein (CRP), erythrocyte sedimentation rate (ESR), fibrinogen, D-dimer, CRP-albumin ratio (CAR), CRP-lymphocyte ratio (CLR), and CRP-monocyte ratio (CMR)] and their diagnostic efficacy. 254 patients were divided into four groups: an obese group (n = 59) and a non-obese group (n = 195) according to their BMI; each of these groups was further divided into a PJI group and an aseptic failure (AF) group. Although biomarker levels were significantly (*p<0.001) higher in patients with PJI compared with patients with AF in both the obese and non-obese groups, no differences were observed between obese and non-obese groups. However, the CRP biomarker showed the greatest diagnostic value in obese patients (AUC=0.982); while the CAR biomarker showed the greatest diagnostic value in non-obese patients (AUC=0.935). Therefore, these findings conclude that obesity does not alter biomarker levels, but it does alter the required diagnostic thresholds.

Microorganisms that inhabit the human digestive tract affect enteric health and disorders (Iqbal et al., 2021). Infection of the intestinal tract with an increasingly recognized range of bacterial pathogens can profoundly alter intestinal function, with or without causing overt diarrhea, which has profound effects on intestinal absorption, nutrition and development, as well as on global mortality (Petri et al., 2008), resulting in more than 1.5 billion cases of enteric-related infections (Iqbal et al., 2021). In the study by Zhang et al., the patterns and trends of enteric infections between 1990 and 2021 in 204 countries were analyzed for health policy formulation, medical resource allocation, and optimization of patient care plans. This study revealed a significant burden on global public health. Although standardized prevalence, incidence, mortality, and disability-adjusted life years (DALYs) rates decreased slightly, the most affected groups were women, children under 15 years of age, and the elderly, especially in regions with low socio-demographic index (SDI). In highly developed regions, the disease burden of typhoid fever declined, with unsafe waters identified as the leading risk factor globally in both 1990 and 2021; while rotavirus was the leading cause of death and DALYs. These findings highlight the need for innovative, targeted preventive healthcare strategies to reduce this global burden of disease.

Chronic hepatitis B virus (HBV) infection is a major public health problem, causing considerable morbidity and mortality, with approximately 296 million people chronically infected and more than 820,000 deaths worldwide. Although morbidity and mortality from HBV infection are liver-related, HBV has been considered non-cytopathic and liver injury is mediated primarily by T cells of the immune system (Seto et al., 2018; Jeng et al., 2023). In the study by Meng et al., the efficacy of blocking the Programmed Death-Ligand 1 (PD-L1) protein to restore the humoral immune response against HBV was evaluated in a murine model. Treatment with anti-PD-L1 was observed to significantly enhance the differentiation of T follicular helper (Tfh) cells and germinal center (GC) B cells, promoting viral clearance. Furthermore, it was observed that plasmacytoid dendritic cells (pDCs) showed the ability to induce immune tolerance and viral persistence, an effect reversed by PD-L1 blockade. These findings point to the PD-L1/pDC axis as a promising therapeutic target in the management and treatment of chronic HBV infection. On the other hand, exosomes are cell-derived nanovesicles that participate in various biological functions, such as the intercellular transport of materials (Liang et al., 2021). Exosomes are associated with immune response, various diseases such as cardiovascular, neurological, and cancer, as well as viral pathogenicity. In this sense, exosomes offer a window into altered states of cells or tissues, and their detection in biological fluids potentially offers a multi-component diagnostic readout (Kalluri and LeBleu, 2020). Exosomes have been shown to be closely involved in the processes of HBV replication and transmission. The mini review by Yuan et al. describes the production process, composition and function of exosomes. Furthermore, the authors delve into the essential role of exosomes in the replication, transmission, and pathological processes of HBV. Therefore, exosomes can act as biomarkers for early detection and have therapeutic potential in the treatment and prevention of HBV.

Central line-associated bloodstream infections represent a significant burden on healthcare, increasing morbidity, mortality, hospitalization time, and associated costs (Odada et al., 2023). A central line-associated bloodstream infection (CLASBI) is defined as a pathogen detected in a blood culture from a patient who had a central line at the time of infection or up to 48 hours before it occurred (Haddadin et al., 2022). In the study by Li et al., the CLABSI pathogenic bacteria characteristic in intensive care unit (ICU) patients were analyzed, and the value of procalcitonin (PCT), lymphocyte ratio (NLR), and platelet to lymphocyte ratio (PLR) in predicting early infections was evaluated. Of 926 patients, 7.88% (73 patients) developed CLABSI, with a predominance of Gram-positive bacteria (60.5%), which showed high resistance to various antibiotics, such as penicillin, erythromycin, clindamycin and oxacillin; but showed sensitivity to vancomycin, linezolid and tetracycline. Peripheral blood levels of PCT, NLR, and PLR were significantly (*p<0.05) higher in infected patients, with PCT being the best individual predictor (AUC=0.856). The combination of the three markers achieved an AUC of 0.917, demonstrating significant utility in the early detection and clinical management of CLABSI. These findings demonstrate that CLABSI in the ICU requires careful attention, as Gram-positive bacteria are the main pathogens, and targeted antibiotic therapy based on microbial characteristics is essential for effective treatment. Furthermore, elevated blood levels of NLR, PLR, and PCT can facilitate the diagnosis of CLABSI, guiding early intervention and improving outcomes.

Periodontitis is a multifactorial chronic inflammatory disease associated with the accumulation of dental plaque (biofilm). It is primarily characterized by the progressive destruction of dental supporting tissues. Periodontitis involves the interaction between bacteria, the host immune response, and environmental factors (Kwon et al., 2021), all of which can influence the development and severity of the disease. These factors can be local or systemic, or non-modifiable, such as sex, age, ethnicity, or genetic factors; and modifiable, such as smoking, stress, obesity, and uncontrolled diabetes mellitus (DM) (Moreno Caicedo et al., 2018). DM is a chronic multifactorial disease triggered by various genetic and/or environmental factors (Artasensi et al., 2020). Type 2 diabetes mellitus (T2DM) is the most common type of DM (Majety et al., 2023), characterized by a progressive loss of insulin secretion from β cells, often in the context of insulin resistance (Artasensi et al., 2020). Diverse studies have associated T2DM with periodontitis, suggesting a bidirectional association between both pathologies (Santos et al., 2015; Turner, 2022). On the other hand, COVID-19 disease is caused by SARS-CoV-2, which has caused respiratory illnesses, complications and deaths worldwide (Zhang et al., 2023). Emerging evidence suggests that individuals with underlying comorbidities associated with COVID-19 (Sanyaolu et al., 2025), such as T2DM (Abdi et al., 2020) and periodontitis (Hernández-Vigueras et al., 2021), are at increased risk of disease susceptibility, leading to more severe clinical presentation and even death.

Regarding the association between periodontitis, T2DM, and COVID-19, there is currently insufficient scientific literature on the relationship between these three diseases. In the study by Muñoz-Carrillo et al., an explanation is provided regarding the relationship between these three pathologies, hypothesizing that the three diseases share important cofactors, which are centered on three main axes: 1) a clinicopathological axis; 2) an axis associated with glycemia; and 3) an immune axis associated with inflammation. Clinicopathological axis: T2DM increases susceptibility to developing periodontitis, especially in severe forms, while poor diabetes control is associated with increased morbidity and mortality from COVID-19. Furthermore, periodontitis can worsen the course of COVID-19, increasing the risk of complications such as hospitalization, ventilator support, and death. Axis associated with glycemia: hyperglycemia in patients with T2DM promotes inflammatory mechanisms that worsen insulin resistance and periodontal tissue destruction. Hyperglycemia also facilitates SARS-CoV-2 infection by altering the expression of enzymes that help the virus infect and replicate, exacerbating the inflammatory response. Immune axis associated with inflammation: chronic inflammation is the common denominator between the three diseases. Periodontitis and T2DM generate an exacerbated inflammatory response, characterized by the overproduction of proinflammatory cytokines (such as TNF-α, IL-1β and IL-6) and activation of immune cells. In COVID-19, this inflammation is amplified, causing cytokine storms and tissue damage, which worsens the clinical course of all three pathologies.

Bovine respiratory disease (BRD) and diarrhea are multifactorial diseases (Ferraro et al., 2021; Song et al., 2022), caused mainly by pathogens such as bacteria and viruses (Gandhi et al., 2023), which infect the lower respiratory tract of cattle, with a high morbidity and mortality rate (Urie et al., 2018). Disease control can prevent morbidity and mortality; however, current scientific evidence on the effectiveness of these control practices in achieving this goal is limited (Sanguinetti et al., 2025). The complexity of infectious diarrhea in calves limits the effectiveness of vaccination. In this regard, trained immunity, which involves immunological memory in innate cells following exposure to pathogens or their antigens to provide increased protection against subsequent exposure to homologous or heterologous infections, offers a promising strategy for preventing unpredictable infections in young animals (Netea et al., 2020). Yeast β-glucan, a cell wall component of fungi (Han et al., 2020), can induce immune memory in innate immune cells (Goh et al., 2023). It has been studied as a prebiotic in animal diets, showing positive effects such as weight gain and reduced diarrhea in calves (Ding et al., 2019; Pornanek and Phoemchalard, 2021). β-glucan has also been observed to stimulate macrophages and immune cells in goats and calves (Angulo et al., 2020). However, its efficacy in preventing diarrhea and BRD in calves is not yet clearly established. In the study by Yan et al., the prophylactic effect of intraperitoneal injection of yeast β-glucan in Holstein calves during their first 74 days of life was evaluated. In 52 healthy newborn Holstein calves, the treatment induced an initial inflammatory response with increased cytokines (IL-1β, IL-6, and TNF-α), immunoglobulins (IgG and IgM), and defensins, improving innate immunity without affecting growth or feed efficiency. Treated calves showed a lower incidence of diarrhea and BRD, especially between 31 and 60 days, as well as a healthier gut microbiota with a higher abundance of *Bifidobacterium*. These results suggest that β-glucan is a promising strategy to prevent infectious diseases in calves.

Sepsis is a serious organ dysfunction caused by an altered immune response to an infection (Cecconi et al., 2018). In its most severe form, sepsis manifests as a drop in blood pressure, which decreases tissue perfusion pressure and causes the hypoxia characteristic of septic shock (Srzić et al., 2022). Sepsis affects more than 30 million people each year and is one of the leading causes of death in critically ill patients worldwide. Furthermore, the cost of sepsis treatment is also the highest among all treatments for the disease (Rocheteau et al., 2015). Despite advances in treatment, its incidence and mortality continue to increase, and its diagnosis and management remain a challenge for healthcare professionals. This is because sepsis is a complex process that affects multiple organs and goes beyond a simple inflammatory or immune response. Its pathogenesis involves inflammatory imbalances, immune dysfunction, mitochondrial damage, coagulopathy, neuroendocrine disorders, endoplasmic reticulum stress, autophagy, and other mechanisms, culminating in organ dysfunction (Huang et al., 2019). In this context, biomarkers for diagnosing sepsis may allow for early intervention that can reduce the risk of death (Faix, 2013). In the study by Chen et al., the potential of S100A8/A9 and resistin as biomarkers to predict mortality in patients with sepsis was evaluated. Serum samples were collected and analyzed from 141 adult sepsis patients (discovery cohort), 43 non-sepsis intensive care units (ICU) patients, 15 healthy volunteers, and 55 sepsis patients along with 17 non-sepsis ICU patients (validation cohort). It was observed that the concentrations of S100A8/A9 and resistin in sepsis patients were noticeably increased relative to non-sepsis patients and healthy controls. In patients with sepsis, elevated serum concentrations of S100A8/A9 (≥ 377.53 ng/mL) were associated with higher survival. In the ICU, the AUC for S100A8/A9 to predict 28-day mortality was 0.617 (*p = 0.032*; 95% CI: 0.513-0.721), and for the organ failure assess (SOFA) score it was 0.750 (*p<0.0001*; 95% CI: 0.660-0.840). In the validation cohort, the AUC were 0.708 (*p=0.032*; 95% CI 0.563–0.854) for S100A8/A9 and 0.698 for SOFA (*p=0.025*; 95% CI 0.550–0.845). On the other hand, in patients with sepsis, serum resistin concentrations were higher in infections caused by gram-negative bacteria than in gram-positive bacteria. Furthermore, resistin levels were predictive of mortality in normal (AUC = 0.810 [*p = 0.034*; 95% CI 0.605–1.00]) and mixed (AUC = 0.708 [*p=0.015*; 95% CI 0.571–0.846]) phenotypes with hyperinflammation. In the normal phenotype, high levels of resistin (≥ 63.695 ng/mL) were associated with lower survival, while in the mixed phenotype with hyperinflammation, elevated levels (≥ 107.64 ng/mL) were related to higher survival. These findings suggest that S100A8/A9 and resistin could be useful for early diagnosis and risk stratification in sepsis, improving clinical decisions in the ICU.

*Mycoplasma pneumoniae* (MP) is an important bacterium (Waites and Talkington, 2004) that causes respiratory tract infections in children and adults, the severity of which can range from mild to life-threatening (Waites et al., 2017). MP is the pathogen of human bronchitis and atypical pneumonia and is called community-acquired pneumonia (Hu et al., 2023), which can cause acute inflammation of the upper and lower respiratory tract, as well as extrapulmonary syndromes (Jiang et al., 2021), in the skin, brain, kidney, musculoskeletal, digestive system, and even blood system after infection (Hu et al., 2023). Although macrolides are the first-line treatment for MP pneumonia, persistent fever and/or clinical deterioration can sometimes complicate treatment and even lead to severe systemic disease. There is no consensus on the optimal alternatives, doses, or duration of treatment for severe cases. However, tetracyclines and fluoroquinolones have been used as second-line treatments with favorable clinical outcomes in children (Ding et al., 2024). Respiratory symptoms caused by MP result from several pathogenetic mechanisms, such as adhesion to host cells, direct damage (such as cytotoxicity and invasion), immune damage induced by inflammatory response, and immune evasion (Jiang et al., 2021; Hu et al., 2023). In the study by Chen et al., the role of B lymphocytes in the immune response of 202 children diagnosed with MP was analyzed by evaluating the CDR3 repertoires of the B cell receptor (BCR) heavy chain. A significant increase in B lymphocytes and elevated levels of inflammatory markers, such as C-reactive protein (CRP), interleukin (IL)-6, and ferritin, were observed, indicating an active immune response. Immunoglobulin levels were elevated in several patients, indicating immune fluctuations during infection. BCR repertoire analysis revealed increased diversity and altered clonotype distribution in MP patients, with preferential usage of IGHV1-18, IGHV7-4-1, and IGHJ6. MP patients showed a bimodal distribution of CDR3 length, with significantly longer CDR3 regions and clonal expansion with 68 unique MP clonotypes. These findings highlight alterations in the BCR repertoire as key to immunity against MP and potential therapeutic targets.
